# Statistical Structural Analysis of Familial Spontaneous Epileptic Cats Using Voxel-Based Morphometry

**DOI:** 10.3389/fvets.2018.00172

**Published:** 2018-07-24

**Authors:** Yuji Hamamoto, Daisuke Hasegawa, Yoshihiko Yu, Rikako Asada, Shunta Mizoguchi, Takayuki Kuwabara, Masae Wada, Aki Fujiwara-Igarashi, Michio Fujita

**Affiliations:** Department of Clinical Veterinary Medicine, Nippon Veterinary and Life Science University, Musashino, Japan

**Keywords:** amygdala, cats, epilepsy, hippocampus, magnetic resonance imaging, voxel-based morphometry

## Abstract

Voxel-based morphometry (VBM) based on high resolution three-dimensional data of magnetic resonance imaging has been developed as a statistical morphometric imaging analysis method to locate brain abnormalities in humans. Recently, VBM has been used for human patients with psychological or neurological disorders such as Alzheimer's disease, Parkinson's disease, and epilepsy. Traditional volumetry using region of interest (ROI) is performed manually and the observer needs detailed knowledge of the neuroanatomy having to trace objects of interest on many slices which can cause artificial errors. In contrast, VBM is an automatic technique that has less observer biases compared to the ROI method. In humans, VBM analysis is performed in patients with epilepsy to detect accurately structural abnormalities. Familial spontaneous epileptic cats (FSECs) have been developed as an animal model of mesial temporal lobe epilepsy. In FSECs, hippocampal asymmetry had been detected using three-dimensional magnetic resonance (MR) volumetry based on the ROI method. In this study, we produced a standard template of the feline brain and compared FSECs and healthy cats using standard VBM analysis. The feline standard template and tissue probability maps were created using 38 scans from 14 healthy cats. Subsequently, the gray matter was compared between FSECs (*n* = 25) and healthy controls (*n* = 12) as group analysis and between each FSEC and controls as individual analysis. The feline standard template and tissue probability maps could be created using the VBM tools for humans. There was no significant reduction of GM in the FSEC group compared to the control group. However, 5/25 (20%) FSECs showed significant decreases in the hippocampal and/or amygdaloid regions in individual analysis. Here, we established the feline standard templates of the brain that can be used to determine accurately abnormal zones. Furthermore, like MR volumetry, VBM identified morphometric changes in the hippocampus and/or amygdala in some FSECs.

## Introduction

Voxel-based morphometry (VBM) based on high-resolution three-dimensional (3D) data of magnetic resonance imaging (MRI) has been established as a statistical morphometric imaging analysis to accurately locate structural brain abnormalities in humans (1). Traditional techniques to evaluate regional brain volume use the region of interest (ROI) method. The ROI method involves tracing the object manually in many slices leading to bias (2). VBM analysis was used in some animal models, such as mice (3), rats (4), baboons (5), and macaques (6). Dogs were also used as human models of aging in VBM studies (7). In addition, gray matter (GM) reduction was found in dogs with idiopathic and structural epilepsy using VBM analysis (8). Historically, many studies used cats as an experimental animal to investigate the function and diseases of the brain. Despite the fact that the physiological information of the feline brain has been elucidated comparatively, VBM analysis has not been performed in cats yet.

In epilepsy, the epileptogenic zone is defined as “the minimum amount of cortex that must be resected (or completely disconnected) surgically to produce seizure freedom” (9, 10) and is the most important aspect of presurgical examinations. The epileptogenic zone is estimated by the following five abnormal cortical zones: (i) the irritative zone (area of cortex which generates interictal spikes); (ii) the seizure-onset zone (area of cortex that initiates clinical seizures); (iii) the symptomatogenic zone (area of cortex which, when activated, produces the initial ictal symptoms or signs); (iv) the structural abnormal zone also known as the epileptogenic lesion (macroscopic lesion which is causative of the epileptic seizures because the lesion itself is epileptogenic or by secondary hyperexcitability of adjacent cortex); and (v) the functional deficit zone (area of cortex that is not functioning normally in the interictal period) (10). In humans, mesial temporal lobe epilepsy (MTLE) is the most common refractory (drug-resistant) epilepsy with focal (limbic) epileptic seizures and hippocampal and/or amygdaloid (mesial temporal lobe) sclerosis. Since patients with MTLE achieve seizure freedom by surgical resection of the sclerotic hippocampus and amygdala selectively, it is considered that the epileptogenic zone of MTLE exists in these regions. In human structural MRI, hippocampal sclerosis can be detected as atrophy with hyperintensity on T2-weighted and/or fluid attenuated inversion recovery (FLAIR) sequences that correlates with histopathological changes including, neuronal loss, gliosis, and atrophy of the pyramidal cell layer. There is strong evidence that cats with temporal lobe epilepsy show hippocampal sclerosis and it is thought that cats with recurrent seizures with orofacial involvement are more likely to have hippocampal pathologies that are detectable by MRI, especially on FLAIR sequences (11).

In previous studies, we reported familial spontaneous epileptic cats (FSECs) as a natural occurring genetic animal model of human MTLE (12–17). The seizure types of FSECs are spontaneous focal limbic seizures with or without evolving generalizations and vestibular stimulation-induced generalized seizures. The epileptogenic zone of FSECs was estimated to be in the amygdala and/or hippocampus using scalp electroencephalography to evaluate the irritative zone (12), video-intracranial electroencephalography to determine the symptomatogenic zone and the seizure-onset zone (13), and interictal and early postictal diffusion and perfusion MRI to detect the seizure-onset and the functional deficit zones (14, 15). Additionally, 3D MR volumetry using the ROI method showed asymmetric hippocampal volume in FSECs as the structural abnormal zone (16). Histologically, the hippocampus and amygdala of FSCEs show decreased neuronal cell numbers compared to normal healthy controls (17).

Based on these findings, we hypothesize that FSECs would show reduction of the hippocampus and/or amygdala that is suspected as the epileptogenic zone in standard VBM analysis. However, standard VBM analysis requires a standard template and tissue probability maps. Therefore, the aims of this study were: (i) to create a feline standard template and tissue probability maps; and (ii) to investigate whether standard VBM analysis can detect the suspected epileptogenic zone of FSECs.

## Materials and methods

### Ethics statement

All animal use procedures were in accordance with Act on Welfare and Management of Animals, and Standards relating to the Care and Keeping and Reducing Pain of Laboratory Animals. The current study, including the maintenance of the FSEC colony, was approved by the Animal Care and Use Committee of Nippon Veterinary and Life Science University (accession nos. 13-24, 26K-29, 26K-27, 27K-8, 27K-10, 28K-2, 28K-4, 29K-4, 29K-5; the representative researcher was D.H.).

### Animal preparation

#### Development of the feline standard template

A total of 38 MRI scans were acquired from 14 healthy cats (7 males and 7 females) aged 6 months to 10 years, to avoid aging changes, to produce the feline standard template. The median age and body weight (range) of these healthy cats were 40 months (16–73 months) and 3.5 kg (2.4–6.0 kg), respectively.

#### VMB analysis

To perform VBM analysis, MRI scans were also acquired from 25 FSECs (14 males and 11 females) and 12 healthy control cats (6 males and 6 females). The median (range) age of FSECs and control cats were 73 months (10–98 months) and 35 months (23–51 months), respectively. The median (range) body weight of FSECs and control cats were 3.6 kg (2.0–5.2 kg) and 3.3 kg (2.6–5.0 kg), respectively.

### Data processing

#### MRI scanning protocol

All MR images were obtained with a 3.0-Tesla unit (Signa® HDtx 3.0T, GE Healthcare, Tokyo, Japan) using 8 ch human knee array radio frequency coil. All the cats were fasted for 12 h prior to MRI scanning. The cats were anesthetized with propofol (7 mg/kg, intravenous) for tracheal intubation and were then maintained with isoflurane (2.0%) and oxygen. During scanning, the cats were positioned in prone with their heads placed in the coil. The cats were infused with 5 mg/kg/h lactate ringer solution and mechanically ventilated with a respiratory rate of 12 breaths/min during MRI. The heart rate was continuously monitored with an MR peripheral gate system and the body temperature was maintained at 36–38°C using warm-water bags placed around the body. In VBM analysis, 3D T1-weighted images were acquired in the sagittal plane using a spoiled gradient recalled acquisition in the steady state sequence (TR/TE = 6.5/3.1 ms, fields of view = 15 × 15 cm, slice thickness = 0.6 mm, slice gap = 0, matrix = 256 × 192, number of excitation = 1, and scan time = 325 s). Additionally, as conventional MRI, fast spin echo (FSE) T1-weighted FLAIR (TR/TE = 2800/8.2–8.4 [auto] ms, FOV = 15 × 15, slice thickness = 3.0 mm, slice gap = 0.5 mm, matrix = 320 × 224, and NEX = 2), FSE T2-weighted (TR/TE = 7000/85 ms, FOV = 15 × 15, slice thickness = 3.0 mm, slice gap = 0.5 mm, matrix = 384 × 288, and NEX = 1), FSE T2-weighted FLAIR images (TR/TE/T1 = 11000/140/2400 ms, FOV = 15 × 15, slice thickness = 3.0 mm, slice gap = 0.5 mm, matrix = 256 × 192, and NEX = 2), and post contrast FSE T1-weighted FLAIR (after intravenous administration of 0.1 mmol/kg gadodiamido) images were obtained in the transverse plane to evaluate hippocampus changes. In addition, FSE 3D-T2 Cube images [TR/TE = 3200/78–90 (auto) ms, slice thickness = 0.6 mm, slice gap = 0 mm, FOV = 15 × 15 cm, matrix size = 512 × 512, NEX = 1] were acquired for hippocampal MR volumetry.

#### Software for VBM

The creation of the feline standard template and the standard VBM analysis were performed using Matlab 6.5 (Mathworks, Natick, Massachusetts) and Statistical Parametric Mapping (SPM) 12 (Wellcome Department of Congnitive Neurology, London, UK) according to a similar standard method for humans (1). In relation to preprocessing of the standard VBM analysis, the normalization was performed using a non-linear transformation and the segmentation used default settings except 30 mm bias full width at half maximum (FWHM) in the SPM12. The smoothing procedure was according to that of dogs (7), which consisted in the smoothing distance in the processing standard template and the preprocessing of standard VBM of 2 and 3 mm FWHM isotropic Gaussian kernel, respectively.

### Preprocessing of VBM analysis

#### Creation of the feline standard template

**Figure 1:**
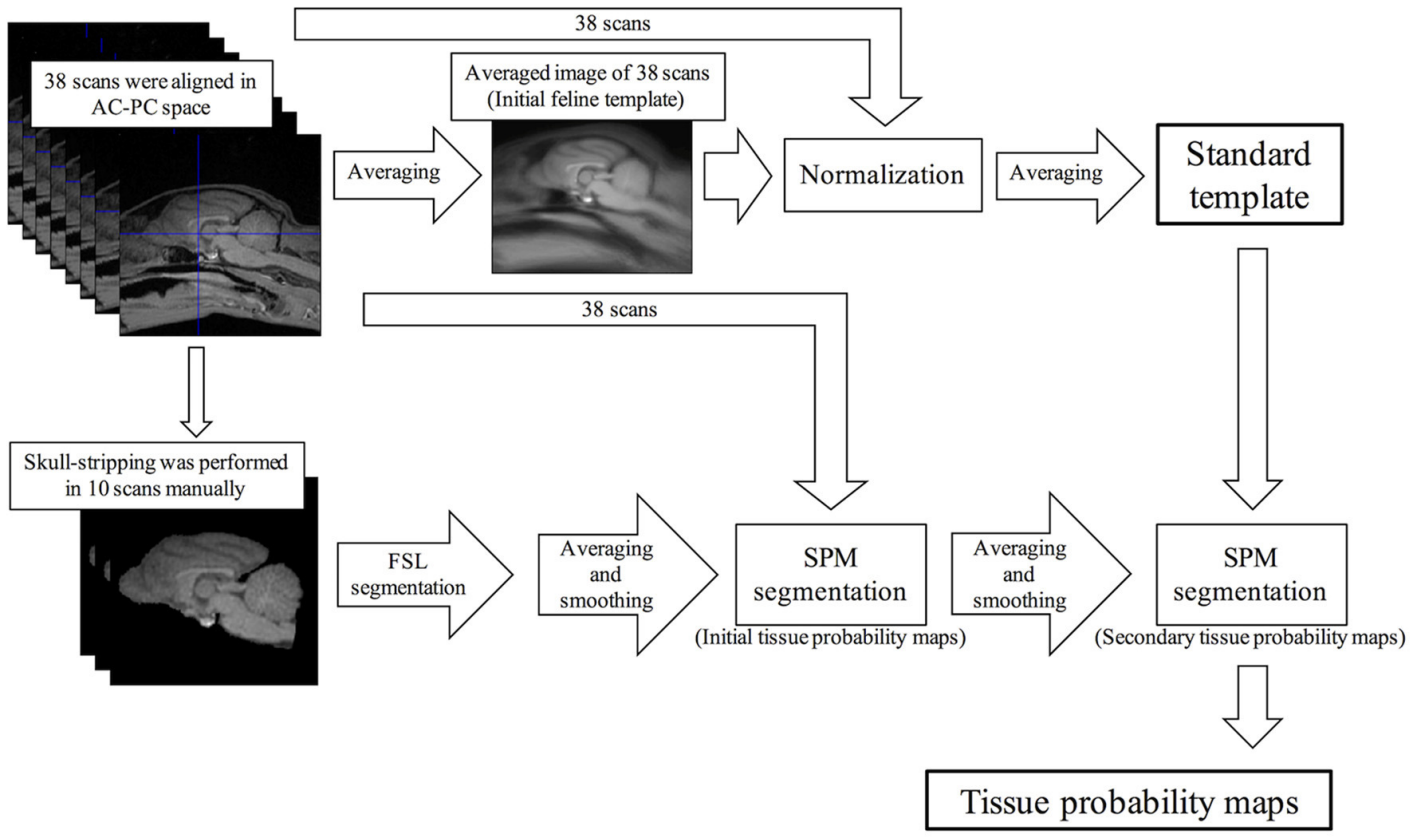
Schematic flow diagrams illustrating the steps of the creation of the feline standard template and tissue probability maps. AC-PC, anterior and posterior commissure; FSL, FMRIB software library; SPM, Statistical Parametric Mapping.

Firstly, we created the feline standard template. A summary of the creation of the feline standard template is shown in Figure 1. A total of 38 scans from 14 healthy cats were aligned in stereotaxic space along the anterior commissure and posterior commissure line. An initial feline template was constructed by an average of these scans. The aligned scans were normalized spatially to the initial feline template and then the normalized scans were averaged. This averaged image was defined as the feline standard template.

In addition, the standard VBM analysis needs tissue probability maps. Of the 38 scans that were used to create the feline standard template, 10 scans were selected randomly except for sex (5 males and 5 females) and skull stripping was performed manually using the MRIcron software (http://people.cas.sc.edu/rorden/mricron/index.html). The skull-stripped scans were segmented into GM, white matter (WM), and cerebrospinal fluid (CSF) using FMRIB software library (FSL) version 5.0.9 (FMRIB, Oxford, UK), and then smoothed to 2 mm FWHM isotropic Gaussian kernel. The initial tissue probability maps were created averaging these smoothed scans of GM, WM, and CSF, respectively. Using these initial probability maps, 38 aligned scans were segmented into GM, WM, and CSF, and then smoothed to 2 mm FWHM isotropic Gaussian kernel, respectively. The tissue probability maps that averaged these images were defined as the secondary probability maps. Finally, the feline standard template was segmented into GM, WM, and CSF using the secondary probability maps. The segmentation errors of the extracranial region on these templates were removed using the MRIcro software. These final images were defined as the feline tissue probability maps.

### VBM analysis

#### Preprocessing of standard VBM

All scans of FSECs (*n* = 25) and healthy control cats (*n* = 12) were aligned in stereotaxic space along anterior commissure and posterior commissure line and normalized to the feline standard template. These scans were segmented into GM, WM, and CSF using the feline tissue probability maps. Since the sample size in this study was small, the segmented scans were smoothed to 3 mm FWHM isotropic Gaussian kernel to perform statistical analysis.

#### Statistical analysis

To compare the differences of 3D T1-weighted imaging in GM between FSECs and controls we used a two-sample *t*-test as a group analysis. For the individual analysis, comparison of 3D T1-weighted imaging in GM between each FSEC and controls was also performed using two-sample *t*-test based on the assumption that the value of FSECs constitutes the mean value of a hypothetical population with a variance equal to that of the controls (18). The initial voxel threshold uncorrected *P* value was set to 0.001 according to a human hippocampal VBM study (19). Clusters were considered as significant when falling below a cluster-corrected *P* [family-wise error (FWE)] = 0.05. The significant regions of significant clusters were identified according to the atlas of the cat brain (20).

### Additional hippocampal MR volumetry

To evaluate the reliability of the VBM analysis, hippocampal MR volumetry based on 3D T2-weighted images was performed using the manual ROI tracing (16) in the hippocampus that showed a significant reduction on individual VBM analysis (see Results). The hippocampal volume was calculated after manual ROI tracing of the hippocampus using the open source OsiriX software (version 9.5; https://www.osirix-viewer.com/).

## Results

**Figure 2:**
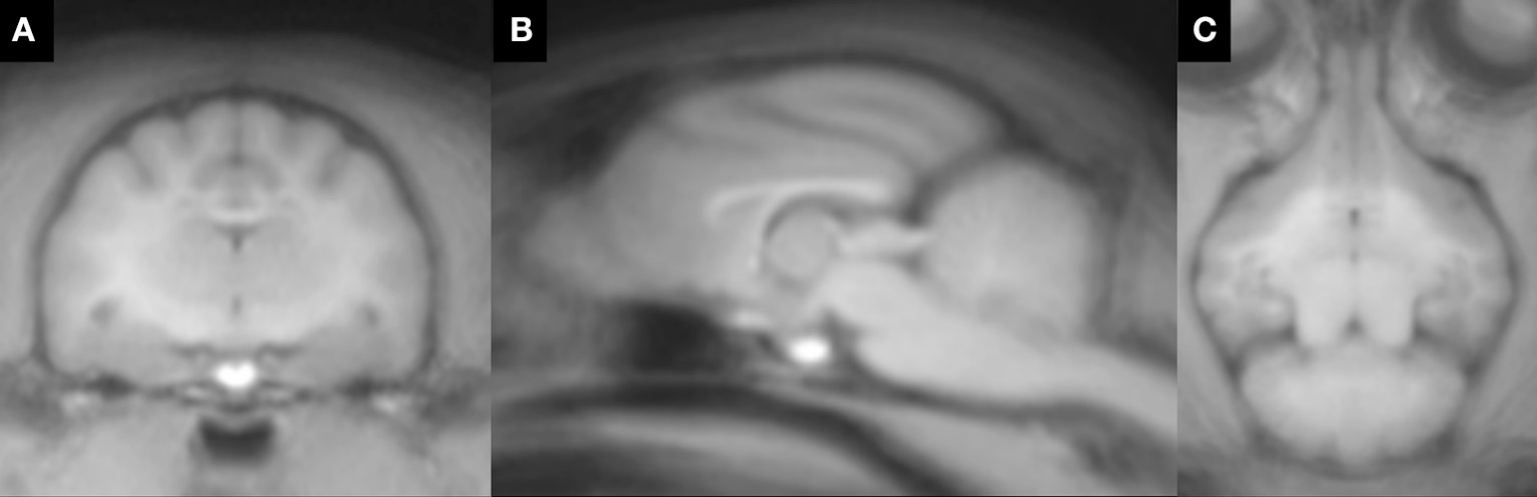
Feline standard template that was created from 38 T1-weighted images. **(A)** transverse plane; **(B)** sagittal plane; **(C)** dorsal plane.

**Figure 3:**
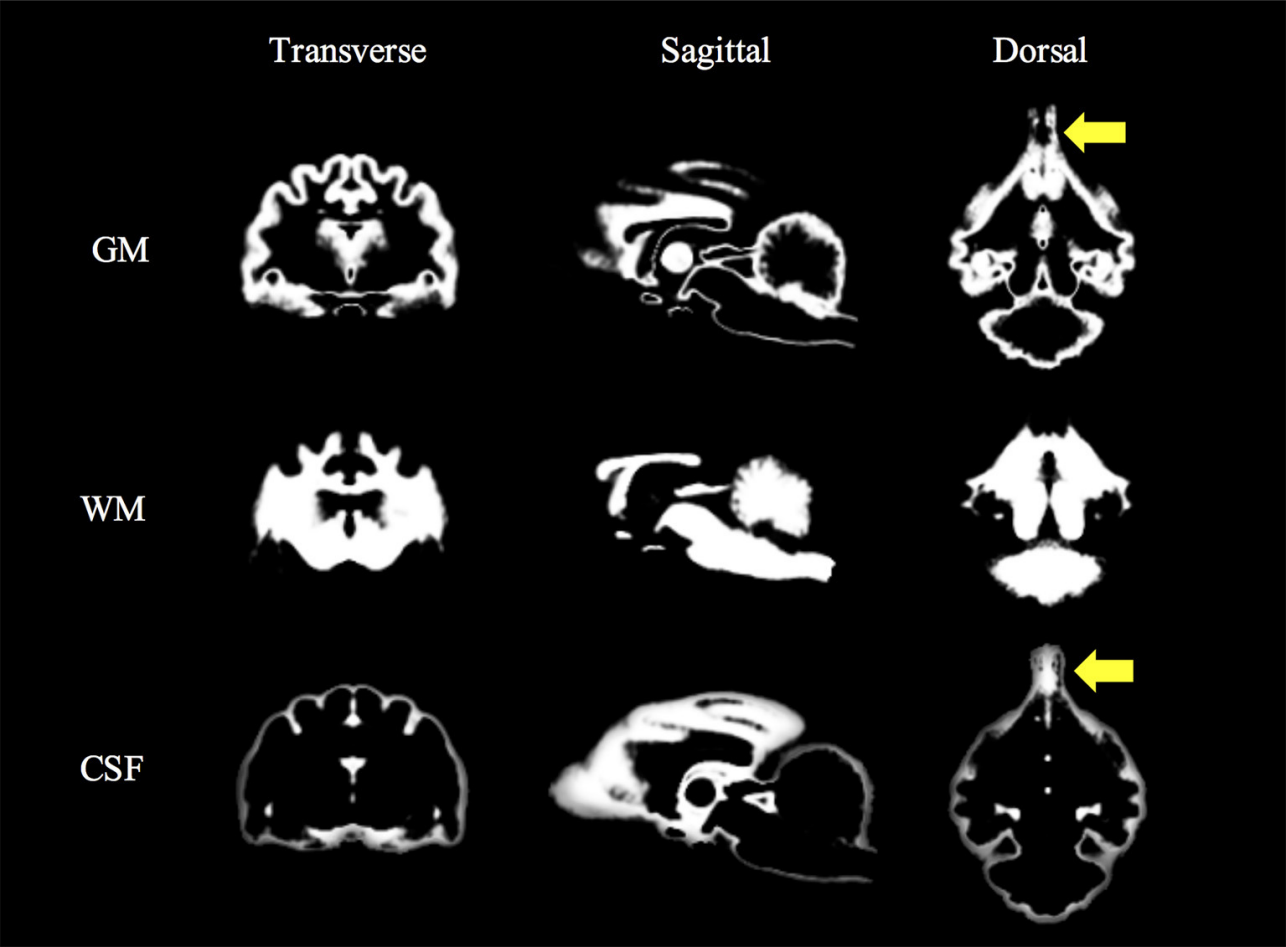
Feline tissue probability maps that were created from 38 T1-weighted images. Yellow arrows (olfactory lobes) indicate the errors of the segmentation. CSF, cerebrospinal fluid; GM, gray matter; WM, white matter.

The feline standard template and tissue probability maps (GM, WM, and CSF) could be created reasonably using the tools and methods for human. However, in the tissue probability maps a part of the olfactory bulb was included in the CSF map by errors of the segmentation. The feline standard template and tissue probability maps are shown in Figures 2, 3, respectively.

**Figure 4:**
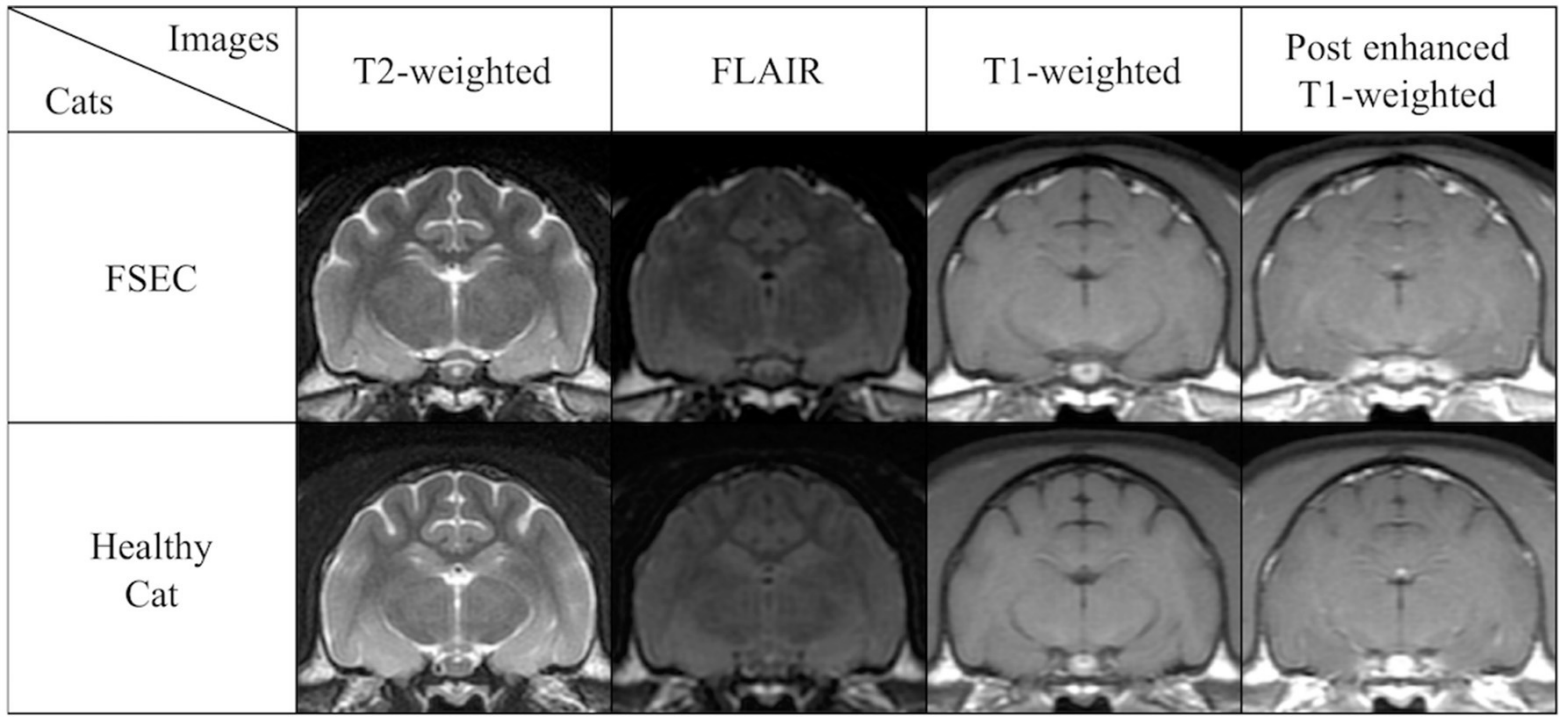
Comparison of conventional MRIs at the level of hippocampus in a typical FSEC (K13MMC: same cat in Figure 5) who showed significant hippocampal and/or amygdaloid reduction on VBM analysis and a healthy cat as a control. The FSEC in this study had no apparent visual differences in hippocampus and amygdala on conventional MRI. FLAIR, fluid attenuated inversion recovery images; FSEC, familial spontaneous epileptic cat; MRI, magnetic resonance imaging.

In visual analysis, FSECs had no remarkable hippocampal changes like hippocampal sclerosis on conventional MRI (Figure 4).

**Figure 5:**
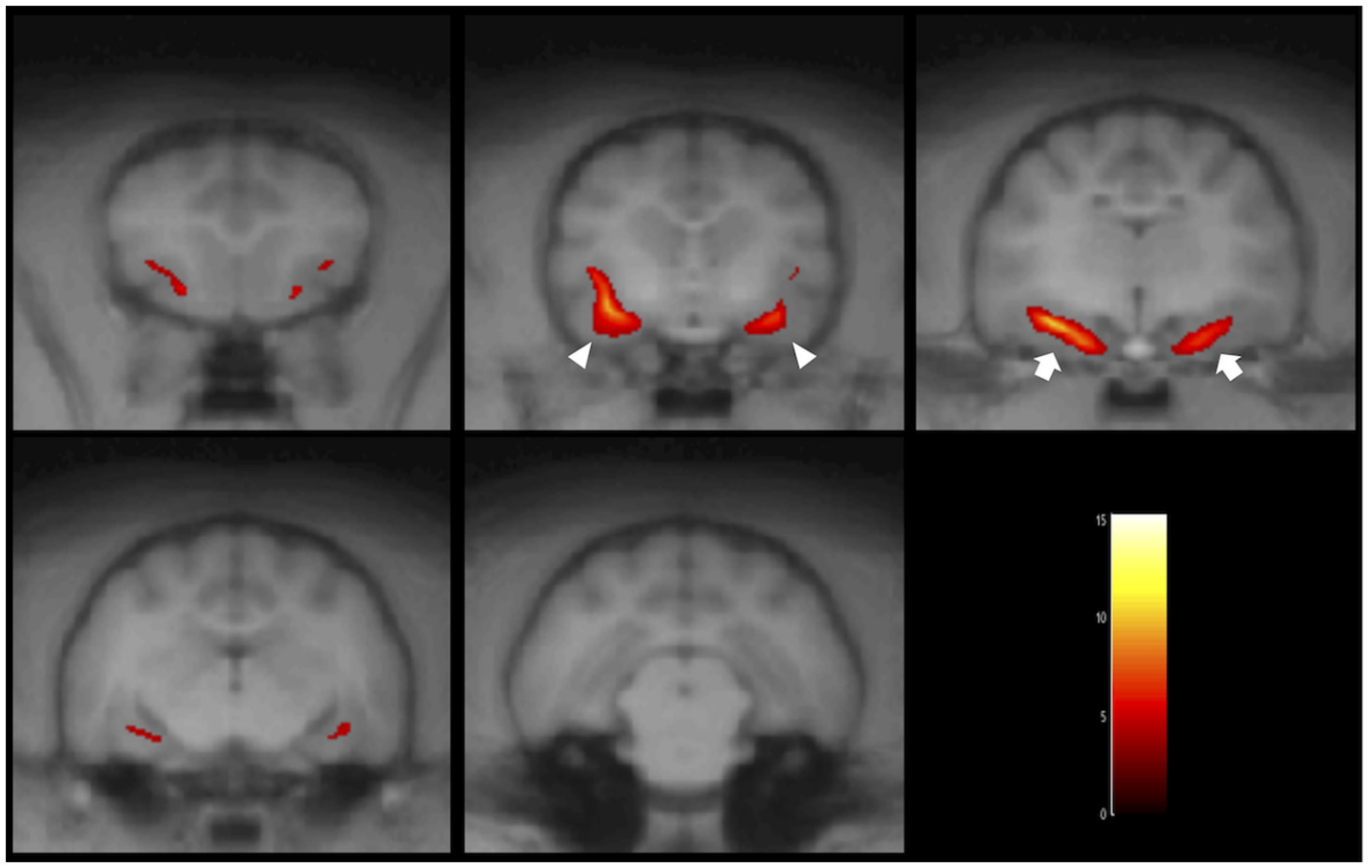
Result of the VBM analysis in a typical FSEC (K13MMC: same cat in Figure 4). The colored regions indicate that cluster-corrected *P*_(*FWE*)_ < 0.05. One FSEC showed decreased hippocampus (white arrows) and amygdala (white arrowheads). FSEC, familial spontaneous epileptic cat; FWE, family-wise error.

When comparing GM between FSEC and control groups using the standard VBM analysis, there were no significant reductions in any regions. However, in the individual analysis, 5/25 (20%) FSECs showed significant hippocampal and/or amygdaloid reduction compared to controls (*P*_(*FWE*)_ < 0.001). In two of these five FSECs, the cluster size of the reduced regions including the hippocampus and/or amygdala was so large that the range of the cluster extended to the cerebral cortex. The results of the five FSECs are shown in Table 1 and the results of the VBM analysis in a typical FSEC is shown in Figure 5. For these five FSECs, hippocampal MR volumetry was performed. The results of hippocampal volumetry of each FSEC are shown in Table 1. The average (± SD) volume of the hippocampi that indicated a significant reduction in VBM analysis was 0.207 cm^3^ (±0.01). These volumes of hippocampi are lower than those of healthy cats reported previously (0.227 ± 0.02 cm^3^) (16).

**Table 1:** Details of the clusters of five FSECs that showed reduction of the hippocampus and/or amygdala in individual analysis.

**Cat no**.	**Cluster level *P_(*FEW*)_* value**	**Cluster size (*k*)**	**Coordinates (mm)**	**Localization**	**Hippocampal volume (cm^3^)**
Y38JFC	< 0.001	225370	−10.2, −2.5, −5.8	Left hippocampus and/or amygdala	0.196
			−16.2, −10.6, −0.1	Left temporal lobe	
			−10.5, −24.1, −0.7	Left cerebellum	
K13MMC	< 0.001	8234	−9.9, −2.5, −5.8	Left hippocampus and/or amygdala	0.217
			−7.2, 5.9, 2,0	Left hippocampus and/or amygdala	
	< 0.001	4778	9.6, −1.6, −5.8	Right hippocampus and/or amygdala	0.214
			12.9, −9.4, −3.4	Right hippocampus and/or amygdala	
			10.8, 2.3, 1.4	Right hippocampus and/or amygdala	
	0.047	1458	1.2, 8.6, −0.1	Bilateral anterior frontal lobe	
D7034	0.001	3477	−10.2, −1.3, −4.0	Left hippocampus and/or amygdala	0.202
			−15.9, −6.4, 2.6	Left hippocampus and/or amygdala	
D7021	< 0.001	284712	−9.9, −2.5, −5.8	Left hippocampus and/or amygdala	0.205
			1.8, 5.6, −0.4	Right frontal lobe	
			−12.3, −11.2, −1.6	Left temporal lobe	
G37IMC	< 0.001	5942	6.9, −28.3, −1.9	Right cerebellum	
			−6.6, −27.1, 1.1	Left cerebellum	
			0.9, −27.4, 5.0	Right cerebellum	
	0.048	1449	−8.7, −1.6, −5.8	Left hippocampus and/or amygdala	0.208

*Cluster size represents the number of significant voxels within each cluster. Coordinates indicate distance from the anterior commissure. FSEC, familial spontaneous epileptic cat*.

## Discussion

In this study, we performed a standard VBM analysis of the feline brain and showed that the feline standard template and tissue probability maps can be created using the VBM tools that have been developed for human. Recently, 7-Tesla MRI-based 3D cortical atlas and tissue probability maps for the domestic cat were also reported (21). We thought that the feline VBM technique was possible most likely due to the comparatively uniform brain shape of this species, differently from dogs which have a large variation of skull type and brain shape among not only breeds but also individuals.

### Creation of the feline standard template and tissue probability maps

In humans, many VBM studies using the SPM software use the Montreal Neurological Institute (MNI) brain as a standard template. The first MNI template (i.e., MNI305) is based on the Talairach brain (22) and was created from an average of 305 T1-weighted MR scans (23). It is known that Talairach coordinates can be transformed to Brodmann area (24). In neurohistology, the Brodmann area is a region of the cerebral cortex categorized according to histological structure and cell organization. Since the structural characteristics correlate with function, it is considered that the Brodmann area indicates regional cerebral function. Therefore, the information of functional anatomy on Brodmann area can be calculated from MNI305 including Talairach coordinates (25). Since the feline standard template in the present study is not normalized to an image like the Talairach coordinates, it is difficult to detect accurate abnormal regions even though we referred to the traditional feline brain atlas (20). Further studies will require normalization to histological coordinates that are correlated with the functional and/or anatomical information to identify abnormal regions accurately. In addition, the feline standard template in the present study was created from a small sample size (*n* = 38) compared to that of humans (NMI305, *n* = 305). Since it is preferable that the templates be created based on many healthy subjects, further feline VBM studies need more MRIs of healthy cats to create a standard template resembling the one of humans. Only then feline VBM analysis may be more adaptable for feline brain disorders and more useful for feline brain research.

This study performed the skull-stripping procedure manually using MRIcron. In humans, the extracranial region can be stripped automatically using the FSL software when tissue probability maps do not exist. Although we tried to perform automatic skull-stripping using FSL, many extracranial regions remained. T1-weighted imaging represents CSF and bone as low intensity. When we performed the skull-stripping procedure manually, it was difficult to distinguish subarachnoid CSF from skull accurately on T1-weighted images. As a consequence, the CSF probability map in the present study may include not only CSF but also bone. Although the manual skull-stripping technique was also used in a previous VBM study of dogs (7), we think that accurate skull-stripping software using automatic technique may be needed for veterinary use.

### Standard VBM analysis for FSECs

This study showed that some FSECs (20%) had a significant structural reduction of the hippocampus and/or amygdala area. Although FSECs in the present study did not show the typical findings of hippocampal sclerosis on the conventional MRI, the regions coincide with suspected epileptogenic zones in FSECs as previously shown (12–15), especially in the hippocampal MR volumetric study (16). Therefore, VBM analysis using 3D T1-weighted imaging may be useful to detect hippocampal abnormality. In veterinary medicine, 3D T1-weighted images are obtained as a conventional sequence in high field MR machines. Therefore, VBM analysis might be introduced to clinical veterinary medicine as well as feline brain research. Although surgery to treat epilepsy has not been established yet in veterinary medicine, individual VBM analysis would be available for presurgical examination of cats with epilepsy. However, to evaluate the epileptogenic zone accurately, it is necessary to synthesize the findings of the individual VBM analysis and other techniques such as seizure video monitoring (seizure semiology), electroencephalography, functional MRI (diffusion, perfusion, T2 map, and blood oxygenation level dependent functional MRI), positron emission tomography, and single photon emission computed tomography.

In this study, the average volume of the hippocampus with a significant reduction on the individual VBM analysis (0.207 cm^3^ ± 0.01) was smaller than that of healthy controls (0.227 cm^3^ ± 0.02) of a previous study (16). The histological study of FSECs (17), showed cell loss in the hippocampus with normal visual findings. Therefore, the individual VBM analysis may be able to detect hippocampal changes which cannot be found visually in MR volumetry. VBM evaluates not only volume but the signal intensity automatically on T1-weighted images. Therefore, it is difficult to evaluate completely its validity using other statistical methods. Since the FSECs used in the present study were not subjected to surgical treatment, such as anterior temporal lobectomy or selective amygdalo-hippocampectomy, the accurate laterality of the epileptogenic zone in each FSEC is unknown. Thus, we think that histological investigations are needed to evaluate the findings of the individual VBM analysis using MRI. Further studies are needed in order to evaluate whether the abnormal regions on the feline VBM show histological changes.

In human familial MTLE, approximately 57–70% patients have hippocampal atrophy (26–28). Kobayashi et al. described that the distribution of the hippocampal atrophy according to the seizure frequency was 46% in patients with seizure remission, 51% in patients with good seizure control under medication, and 100% in patients with refractory MTLE (26). Although we did not take into account the seizure frequency of FSECs in the present study, regarding patient distribution, FSECs may be classified as benign familial MTLE.

We found that there is no significant reduction in GM of FSECs compared to that of controls. Although the hippocampus of FSECs presented asymmetric volume in our previous volumetric study using the ROI method, the hemilateral and bilateral hippocampal volumes did not significantly decrease compared to those of controls (16). In a previous histopathological study of FSECs, neuronal decrease was only observed in CA3 of the hippocampus and the central nucleus of the amygdala and gliosis was found only in CA4, which is equivalent to the “no hippocampal sclerosis and gliosis only” of the international consensus for human hippocampal pathology (17). In other words, FSECs did not show drastic epilepsy-related neuropathological changes such as hippocampal sclerosis, amygdaloid sclerosis, granule cell pathology, and mossy fiber sprouting. Therefore, the finding in the present study may be reliable. Additionally, since human familial MTLE displays a progressive reduction of hippocampal volume independently of seizure frequency (28), the hippocampal reduction in the group analysis might be observed by performing longitudinal (time-course) monitoring and sequential VBM analysis in FSECs.

### VBM methodology

This study was analyzed using the standard VBM technique. In humans, the optimized VBM technique (29) and the diffeomorphic anatomical registration using exponentiated lie algebra-based VBM technique (30) have been developed as advanced methods. However, since these advanced methods require more complicated procedures, they were not performed in the present study. In a VBM methodological study using patients with temporal lobe epilepsy, Keller et al. reported that the hippocampus and some artifact regions were significantly reduced (31). Therefore, it is unknown whether the reduction of other regions, except for hippocampus and/or amygdala, in the present study are the result of artifacts. Further studies are needed to define the normal state of other regions including advanced VBM analysis.

In VBM analysis, modulation is used to correct for changes in brain volume caused by spatial normalization (29). Using the modulation procedure, the global brain volume can be calculated on normalized images. Generally, the brain volume is used as an optional step to correct for effects caused by individual differences in brain size in statistical analysis. Since this study did not use the modulation procedure, we could not perform a corrected statistical analysis using volume. Of the five FSECs that showed hippocampus and/or amygdala as the most significant decreased region in individual analysis, the range of the decreased region in 2 FSECs extended to the cerebral cortex. This finding may be due to overestimation since we did not correct for brain volume. However, Radua et al. reported that the modulation procedure was associated with a substantial decrease in the sensitivity to detect mesoscopic volume abnormalities in the cortical and subcortical regions (32). Therefore, further studies need to evaluate the need of the modulation procedure in feline VBM analysis.

### Use of VBM analysis in feline veterinary medicine

Clinically, it is suspected that hippocampal changes caused by epilepsy in many cats are in the temporal lobe (33). In a histopathological study, Wagner et al. reported that one-third of cats with epilepsy have neuropathological changes resembling human hippocampal sclerosis (34). Since the automatic VBM technique is simple and quick compared to the manual ROI technique, if a more accurate feline standard template with functional and/or anatomical coordinates is established, it would be possible to analyze a large sample size. Thus, it would be interesting to investigate the hippocampal reduction in cats with epileptic seizures using the VBM analysis.

Additionally, VBM analysis is also used in other brain disorders such as Alzheimer's disease and psychopathic disorders. In particular, the diagnosis of Alzheimer's disease is commonly performed using MRI to differentiate it from other brain diseases. In addition, VBM analysis can detect specific atrophy of Alzheimer's disease (35). Recently, Chambers et al. reported that the domestic cat is an attractive model species for Alzheimer's disease to study therapeutic interventions (36). Since cats have a gyrated and sufficient brain volume compared to rodents, that are commonly used as experimental animals for human, the findings of the VBM analysis in cats with Alzheimer's disease may be useful for human studies.

## Author contributions

DH conceived and designed the experiments; YH, YY, RA, SM, and TK performed the experiments; YH analyzed the data and wrote the manuscript; DH, MW, AF-I, and MF performed the critical revisions of the manuscript. All authors approved the final manuscript.

### Conflict of interest statement

The authors declare that the research was conducted in the absence of any commercial or financial relationships that could be construed as a potential conflict of interest.

## Abbreviations

CSF: cerebrospinal fluid
FLAIR: fluid attenuated inversion recovery images
FSE: fast spin echo
FSEC: familial spontaneous epileptic cat
FWE: family-wise error
FWHM: full width at half maximum
GM: gray matter
MNI: Montreal Neurological Institute
MR(I): magnetic resonance (imaging)
MTLE: mesial temporal lobe epilepsy
ROI: region of interest
SPM: Statistical Parametric Mapping
3D: three-dimensional
VBM: voxel-based morphometry.
